# The Actin-Sequestering Protein Thymosin Beta-4 Is a Novel Target of Hypoxia-Inducible Nitric Oxide and HIF-1α Regulation

**DOI:** 10.1371/journal.pone.0106532

**Published:** 2014-10-01

**Authors:** Yun-Kyoung Ryu, Joo-Hyun Kang, Eun-Yi Moon

**Affiliations:** 1 Department of Bioscience and Biotechnology, Sejong University, Seoul, Korea; 2 Molecular Imaging Research Center, Korea Institute of Radiological and Medical Science, Seoul, Korea; University of Dundee, United Kingdom

## Abstract

The actin-sequestering protein thymosin beta-4 (Tβ4) is involved in various cellular and physiological processes such as proliferation, motility, growth and metastasis. Nitric oxide (NO) promotes tumor invasiveness and metastasis by activating various enzymes. Herein, we investigated whether hypoxia-inducible NO regulates Tβ4 expression and cancer cell migration using HeLa cervical cancer cells. NO production and Tβ4 expression were increased in a hypoxic condition. The treatment with N-(β-D-Glucopyranosyl)-N2-acetyl-S-nitroso-D, L-penicillaminamide (SNAP-1), to generate NO, enhanced the transcription of Tβ4 and cancer cell migration. SNAP-1-induced cell migration was decreased by the inhibition of Tβ4 with small interference (si) RNA. In a hypoxic condition, treatment with N^G^-monomethyl-L-arginine (L-NMMA), nitric oxide synthase (NOS) inhibitor, reduced Tβ4 transcriptional activity, and hypoxia-inducible factor (HIF)-1α. Hypoxia-induced cancer cell migration was also decreased by L-NMMA treatment. In a normoxic condition, Tβ4 transcriptional activity was decreased in the cells incubated in the presence of L-NMMA after co-transfection with Tβ4 promoter and GST-conjugated HIF-1α. Collectively, these results suggest that NO could regulate the expression of Tβ4 by direct or indirect effect of HIF-1α on Tβ4 promoter.

## Introduction

Thymosin beta-4 (Tβ4) is a small and naturally occurring 43 amino acid peptide present in all cells except erythrocytes. Tβ4 was initially isolated from the thymus in 1981 [1]. The beta-thymosins including Tβ4, are the most abundant members of highly conserved polar 5 kDa peptides [2]. Lys-38 of the Tβ4 protein is cross-linked to Gln-41, a G-actin monomer leading to the formation of 1∶1 complexes [3]. Tβ4 can also depolymerize F-actin resulting in multiple diverse cellular functions [4]. Tβ4 plays a role in anti-apoptotic response to an external stress [5], paclitaxel-resistance *via* ROS production [6]–[8] and HIF-1α stabilization *via* Erk activation [9]. In addition, Tβ4 regulates cancer cell migration through various signaling pathways [10]–[13]. Tβ4 triggers the epithelial-mesenchymal transition by upregulating integrin-linked kinase [14]. Tβ4 in colon adenocarcinoma plays a role in malignant progression and invasion [15], [16]. However, the mechanism of action on Tβ4 expression in hypoxia conditioning has not been elucidated.

Nitric oxide (NO) is an uncharged free radical and plays a role in vasodilation, neurotransmission and anti-platelet aggregation [17]. NO is synthesized from the amino acid L-arginine by three different isoforms of NO synthase (NOS), nNOS, eNOS and iNOS [18]–[20]. nNOS and eNOS are constitutively expressed [21] and iNOS is induced by endotoxins such as lipopolysaccharide, various cytokines and the HIF-1 mediated pathway [22]. Constitutively generated NO is a mediator of diverse physiological cellular functions [17], which are mediated by the activation of soluble guanylyl cyclase and the production of the second messenger, cyclic guanine monophosphate [21]. NO production in tumor cells is also increased under hypoxic conditions [23]. The NO cellular effect is dependent on cellular concentration leading to DNA damage, cell death and anti-apoptosis [24]. In addition, NO is associated with cancer cell biology including apoptosis, cell cycle, cancer progression and metastasis, angiogenesis, chemoprevention and anticancer therapeutic efficacy [25]. The high NO level in macrophages mediates host defense against bacteria or tumor cells [26]. However, the chronic NO production may contribute to inflammation-associated tissue injury and initiate cancers [27]. NO production in tumor cells inhibits primary tumor growth but promotes tumor invasiveness and metastasis by activating various enzymes [27], [28]. The exposure of NO enhances cell motility in various types of cancer cells [28]–[30]. However, whether hypoxia-inducible NO can affect cancer cell migration and Tβ4 expression could be regulated by NO in cancer cells is unclear.

Herein, we investigated whether Tβ4 expression and cell migration could be regulated by hypoxia-inducible NO in HeLa cervical cancer cells. We found that NO production and Tβ4 expression were increased in a hypoxic condition. The treatment with SNAP-1, to generate NO, also enhanced the transcription of Tβ4 and cancer cell migration, which was decreased by inhibiting Tβ4 expression. N^G^-monomethyl-L-arginine (L-NMMA), nitric oxide synthase (NOS) inhibitor reduced Tβ4 level and hypoxia-induced cancer cell migration. In addition, Tβ4 expression was increased by co-transfection with GST-conjugated HIF-1α, which was inhibited in the presence of L-NMMA. Our data suggest that NO could regulate Tβ4 expression *via* direct or indirect interaction of HIF-1α with the Tβ4 promoter.

## Materials and Methods

### Reagents

Anti-rabbit HIF-1α antibodies were purchased from Santa Cruz Biotechnology (Santa Cruz, CA, USA). Antibodies reactive with actin and α-tubulin were obtained from Sigma-Aldrich (St. Louis, MO, USA). Antibodies reactive with Tβ4 were obtained from R&D Systems (R&D Systems, Minneapolis, MN, USA). L-NMMA was obtained from Sigma-Aldrich. N-(β-D-Glucopyranosyl)-N2-acetyl-S-nitroso-D, L-penicillaminamide (SNAP-1) was purchased from Calbiochem (La Jolla, CA, USA). Except where indicated, all other materials were obtained from Sigma-Aldrich.

### Cell culture

HeLa cells were obtained from the Korea Research Institute of Bioscience and Biotechnology (KRIBB) cell bank (Daejeon, Korea). Cells were maintained and cultured in Dulbecco’s modified Eagle’s medium supplemented with 10% fetal bovine serum (GIBCO, Grand Island, NY, USA), 2 mM L-glutamine, 100 U/mL penicillin and 100 U/mL streptomycin. For the hypoxia conditioning, cells were exposed to hypoxia (0.5% O_2_) for an appropriate time by incubating the cells in an anaerobic incubator (Forma Scientific, Marietta, OH, USA) in 5% CO_2_, 10% H_2_ and 85% N_2_ at 37°C. Then, cells were incubated at 37°C in an atmosphere of humidified normoxia incubator with 5% CO_2_ and 95% air.

To knock-down Tβ4 or HIF-1α expression, HeLa cells were transfected with Tβ4- or HIF-1α-siRNA (Bioneer, Daejeon, Korea) using Lipofectamine 2000 (Invitrogen, Grand Island, NY) and incubated for 24 h. Sequence for siRNA was 5′-ccg ata tgg ctg aga tcg a-3′ in Tβ4 gene and 5′-tga tac caa cag taa cca a-3′ in HIF-1α gene. Then, cells transfected with Tβ4-siRNA were treated with SNAP-1 for the measurement of cell migration and cells transfected with HIF-1α-siRNA were incubated under hypoxic condition to examine Tβ4 expression as below.

### Nitrite measurement

Accumulated nitrites were measured in the cell supernatant using the Griess reaction [31]. Briefly, 100 µL of supernatant from each well were mixed with 100 µL of Griess reagent (0.1% naphthylethylenediamine dihydrochloride and 1% sulfanilamide in 2% phosphoric acid) in 96-well microtiter plates. Absorbance was read at 540 nm using an ELISA reader (Molecular Devices, Sunnyvale, CA, USA).

### Cell migration assay

HeLa cell migration was measured using the methods modified from our previous report [12]. Briefly, when HeLa cell density was confluent in a 35-mm^2^ culture dish (Corning, NY, USA), three wound lines in the form of a cross were made by scratching the cellular layer with a plastic pipette tip. Then, floating cells were rinsed and fresh medium was added. As the incubation progressed, the scratch width narrowed and was recorded by taking photographs under a phase contrast microscope. The empty area in each time point was quantified with NIH image analysis software (version 1.62) and compared with the initiation of cell migration.

### Tβ4 promoter reporter assay

Predesigned Tβ4 (Gene Accession#, NM_021109) promoter sequence of 1,242 bp was obtained from GeneCopoeia Inc. (Rockville, MD, USA). The Tβ4 promoter was cloned into Gaussia luciferase (GLuc) reporter plasmid vector, pEZX-PG02. HeLa cells were transfected with the Tβ4-GLuc plasmid and incubated for the appropriate time. Secreted GLuc was obtained from cell culture medium. Next, conditioned medium was collected after the indicated time intervals and analyzed for the presence of reporter protein using a luminometer (Berthold Technology, Germany) and coelenterazine as a substrate for GLuc according to the manufacturer’s protocol (New England Biolabs, Ipswich, MA, USA).

### Reverse transcriptase polymerase chain reaction (RT-PCR)

Total RNA was isolated from HeLa cells using TRIZOL reagent (Invitrogen, Carlsbad, CA, USA). cDNA was synthesized from 1 µg of total RNA using oligo-dT_18_ primers and reverse transcriptase in a final volume of 21 µL (Bioneer, Taejeon, Korea). For standard PCR, 1 µL of the first-strand cDNA product was used as a template for PCR amplification with Taq DNA polymerase (Bioneer, Taejeon, Korea). PCR amplification proceeded using oligonucleotides specific for human Tβ4 (forward: 5′-atg tct gac aaa ccc gat atg gc-3′, reverse: 5′-tta cga ttc gcc tgc ttg ctt c-3′), HIF-1α (forward: 5′-ctc aaa gtc gga cag cct ca-3′, reverse: 5′-gat tgc ccc agc agt cta ca-3′) and GAPDH (forward: 5′-gaa ggt gaa ggt cgg agt c-3′, reverse: 5′-gaa gat ggt gat ggg att tc-3′). PCR products were detected using a 1.2% agarose gel electrophoresis.

### Western blot analysis

Western blot analysis was performed according to standard protocol. HeLa cells treated in various experimental conditions were harvested and then lysed in ice-cold lysis buffer containing 0.5% Nonidet P-40 (vol./vol.) in 20 mM Tris-HCl at a pH of 8.3; 150 mM NaCl; protease inhibitors [2 µg/mL aprotinin, pepstatin; 1 µg/mL leupeptin; 1 mM phenylmethyl sulfonyl fluoride (PMSF)] and 1 mM Na_4_VO_3_, phosphatase inhibitor. Lysates were incubated for 1 h in ice prior to centrifugation at 13,000 rpm for 10 min at 4°C. Proteins in the supernatant were denatured by boiling for 5 min in sodium dodecyl sulfate (SDS) sample buffer. Sample amount of proteins were separated by 10% SDS-polyacrylamide gel electrophoresis (SDS-PAGE) and then transferred to nitrocellulose membranes by electroblotting. After transferring, equal protein loading was verified by Ponceau S staining. The membranes were blocked with 5% skim milk in Tris-buffered saline with Tween 20 (TBST) (10 mM Tris-HCl, pH 7.6, 150 mM NaCl, 0.5% Tween 20), then incubated with the indicated antibodies. Bound antibodies were visualized with HRP-conjugated secondary antibodies with the use of enhanced chemiluminescence (ECL). Immune-reactive bands were detected using X-ray film.

### Statistical analyses

Experimental differences were tested for statistical significance using ANOVA and Student’s *t*-test. A *p-*value<0.05 was considered to indicate significance.

## Results

### Hypoxia-inducible NO augments Tβ4 expression and cell migration

**Figure 1:**
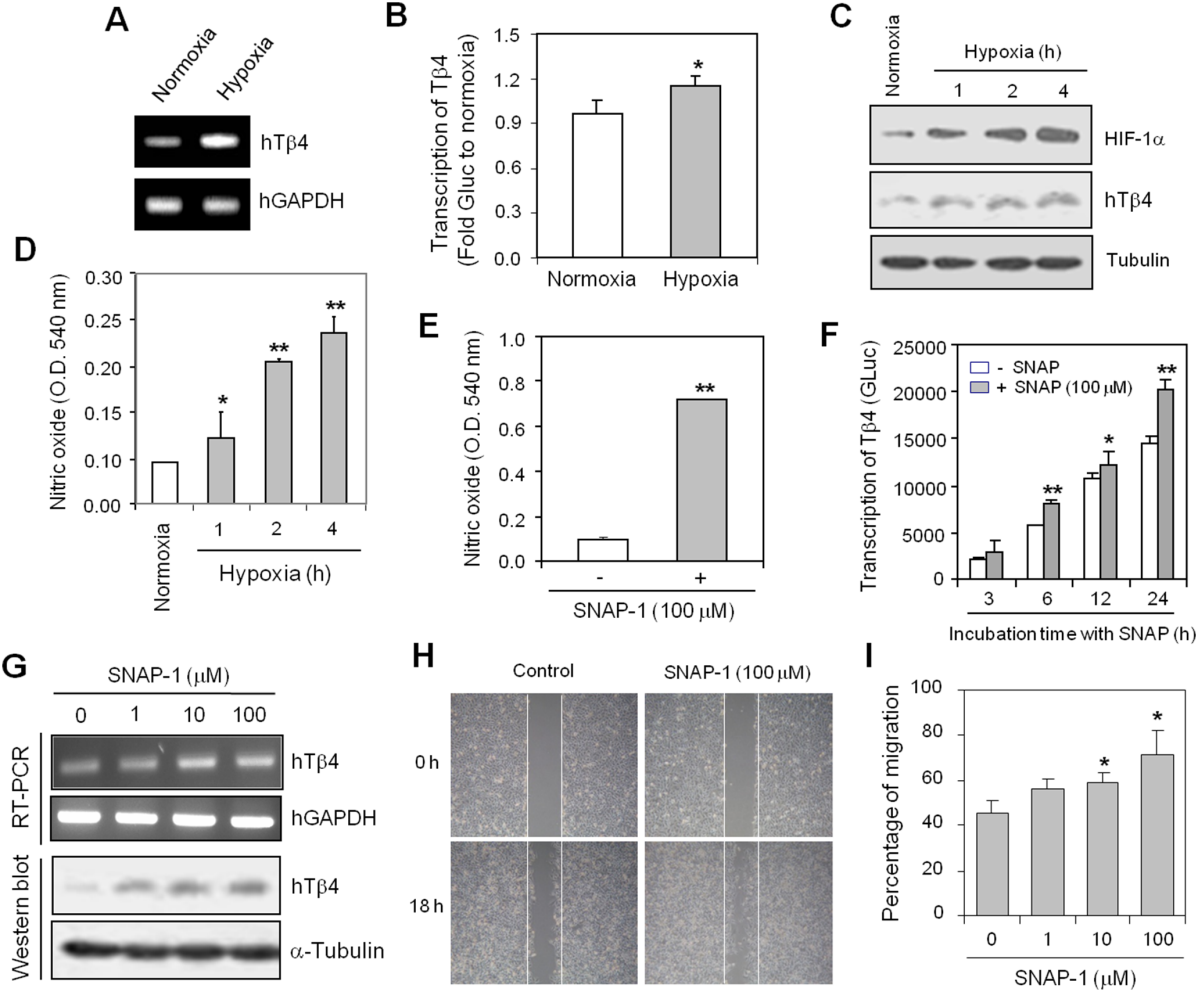
Nitric oxide (NO) enhanced thymosin beta-4 (Tβ4) expression and cell migration. **A:** HeLa cells were incubated under normoxic or hypoxic conditions for 1 h. RNA was purified with TRIZOL reagent as described in the Materials and Methods, and the Tβ4 transcript level was measured using reverse transcriptase polymerase chain reaction (RT-PCR). **B:** HeLa cells were transfected with a Gaussia luciferase plasmid Tβ4 promoter (Tβ4-GLuc) for 12 h and were incubated under normoxic or hypoxic conditions for 1 h. GLuc activity in culture medium was measured with a luminometer using GLuc substrate and represented as fold GLuc to normoxia control. **C–D:** HeLa cells were incubated under normoxia or hypoxia condition for an appropriate time. Cell lysates were prepared and the protein level of HIF-1α and Tβ4 in HeLa cells was detected by Western blot analysis (**C**). NO production was detected with Griess reagents (**D**). Data in the bar graph are means ± standard error of the difference (SED). *p<0.05; **p<0.01, statistical significance *vs*. normoxia control (**B and D**). **E:** HeLa cells were treated with 100 µM SNAP-1 for 12 h. NO production was detected with Griess reagents. Data in the bar graph are means ± SED. *p<0.05, statistical significance in SNAP-1-treated group *vs*. untreated control. **F:** HeLa cells were transfected with Tβ4-GLuc plasmids and treated with 100 µM SNAP-1 for 12 h. GLuc activity in culture medium was measured using a luminometer with a GLuc substrate. Data in the bar graph are means ± SED. *p<0.05; *p<0.01, statistical significance *vs*. Tβ4 promoter activity in the SNAP-1-untreated group at each time point. **G:** HeLa cells were treated with SNAP-1 for 12 h. RNA was purified and cell lysates were prepared from HeLa cells. The Tβ4 level was measured by RT-PCR (**top**) and Western blot analysis (**bottom**). **H–I:** A monolayer of HeLa cells was scratched and incubated for 18 h in the presence or absence of 100 µM SNAP-1. Next, cell migration was photographed with a phase-contrast microscope. Pictures were taken at the same magnification, 200x. Data are representative of four experiments (**H**). The empty area in each SNAP-1 concentration was quantified using NIH image analysis software (version 1.62) and compared with the initiation of cell migration. Percentage of cell migration is presented as a bar graph. Data in the bar graph are means ± SED. *p<0.05, statistical significance *vs*. cell migration in the SNAP-1-untreated control group (**I**).

Because Tβ4 plays a role in cell migration after hypoxia conditioning [12] and NO production is increased under hypoxic conditions [23], we examined whether Tβ4 expression could be regulated by NO production using HeLa cervical cancer cells. The Tβ4 expression level was enhanced by 1 h-incubation under hypoxic condition (Fig. 1A). When cells were incubated under hypoxic or normoxic conditions for 1 h and subsequently incubated under normoxic conditions for 15 h, cell migration in HeLa cervical cancer cells was increased by hypoxia conditioning as compared to that in hypoxia-unexperienced control cells (Fig. S1A and B). Then, we transfected HeLa cells with plasmid containing gaussia luciferase (GLuc) under the control of Tβ4 promoter in order to confirm whether Tβ4 transcription was increased upon hypoxia treatment. Transcription of Tβ4 assessed by gaussia activity of Tβ4 promoter was enhanced under the same experimental conditions (Fig. 1B). Hypoxic condition was confirmed by increased HIF-1α level and Tβ4 protein level was also enhanced under hypoxic conditions (Fig. 1C). The level of HIF-1α and Tβ4 protein in hypoxia-experienced cells was reduced by reoxygenation after 1 h-incubation under hypoxic condition but it was significantly higher than that in control during 15 h (Fig. S1C). Then, to detect NO production under hypoxic conditions, culture-conditioned medium was reacted with Griess reagent. We proved experimentally NO production under hypoxic conditions (Fig. 1D). Next, we examined whether NO regulated directly Tβ4 expression. We used SNAP-1 as the NO donor which is a synthetic chemical reagent that releases NO continuously over a period of time and mimics the physiological action of NO (Fig. 1E). SNAP-1 treatment enhanced transcription of Tβ4 assessed by gaussia activity of Tβ4 promoter in a time-dependent manner (Fig. 1F). SNAP-1 treatment also augmented the level of Tβ4 transcript and protein in a dose-dependent manner (Fig. 1G). To confirm the effect of NO on cell migration, we treated cells with SNAP-1 for 18 h. Results showed that cell migration was increased by the treatment with 1, 10 and 100 µM SNAP-1 (Fig. 1H and I). Data suggest that hypoxia-inducible NO could regulate cell migration *via* Tβ4 expression.

### SNAP-1, NO donor-mediated cell migration is dependent of Tβ4 expression

**Figure 2:**
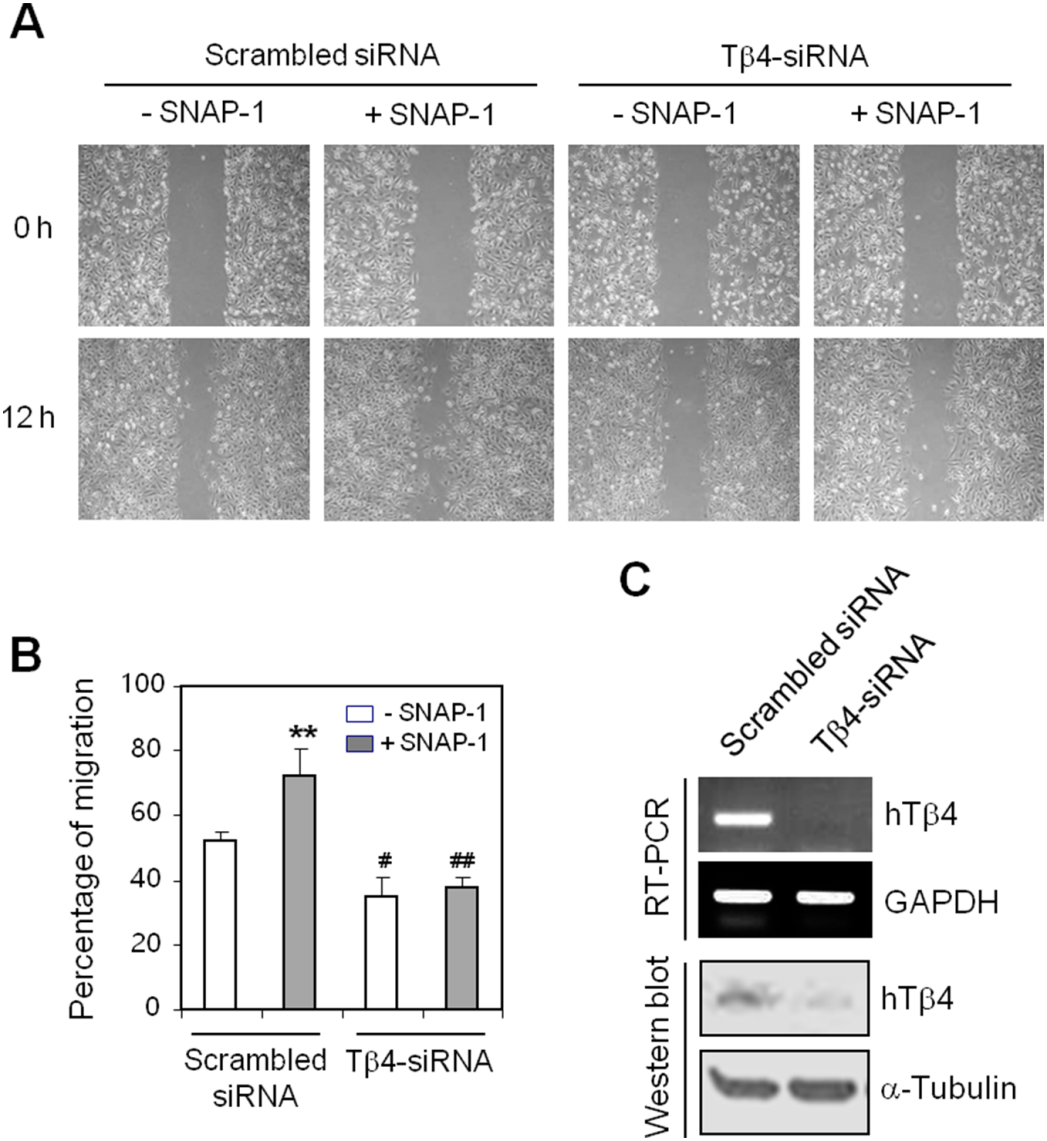
Cell migration increased by SNAP-1 was inhibited by Tβ4 expression. **A–C**: Tβ4 expression in HeLa cells was inhibited by Tβ4-siRNA transfection for 24 h. A monolayer of HeLa cells was scratched and incubated in the presence or the absence of 100 µM SNAP-1 for 12 h. Next, cell migration into the space left by the scratch was photographed with a phase-contrast microscope. Pictures were taken at the same magnification, 200x. Data represent four experiments (**A**). The empty area in each time point was quantified using NIH image analysis software (version 1.62) and compared with the initiation of cell migration. Percentage of cell migration is presented as a bar graph. Data in the bar graph are means ± SED. **p<0.01, statistical significance *vs*. cell migration in SNAP-1-untreated control group. ^#^ *p*<0.05, statistical significance *vs*. cell migration in scrambled-siRNA-transfected and SNAP-1-untreated control group. ^##^ *p*<0.01, statistical significance *vs*. cell migration in scrambled-siRNA-transfected and SNAP-1-treated control group (**B**). RNA was purified with TRIzol reagent as described in the Materials and Methods, and the Tβ4 level was measured by RT-PCR (**C, top**) and Western blot analysis (**C, bottom**).

To examine the effect of NO on cell migration *via* Tβ4 expression in HeLa cells, HeLa cells were transfected with Tβ4-siRNA to inhibit Tβ4 expression and treated with SNAP-1. As shown in Figure 2A and B, HeLa cell migration increased by SNAP-1 was attenuated by Tβ4-siRNA transfection. Inhibition of Tβ4 expression was confirmed by RT-PCR (Fig. 2C, top) and Western blot analysis (Fig. 2C, bottom). Data implicate that NO-mediated cell migration was dependent on Tβ4 expression.

### Hypoxia-induced Tβ4 expression is inhibited by L-NMMA, a NOS inhibitor

**Figure 3:**
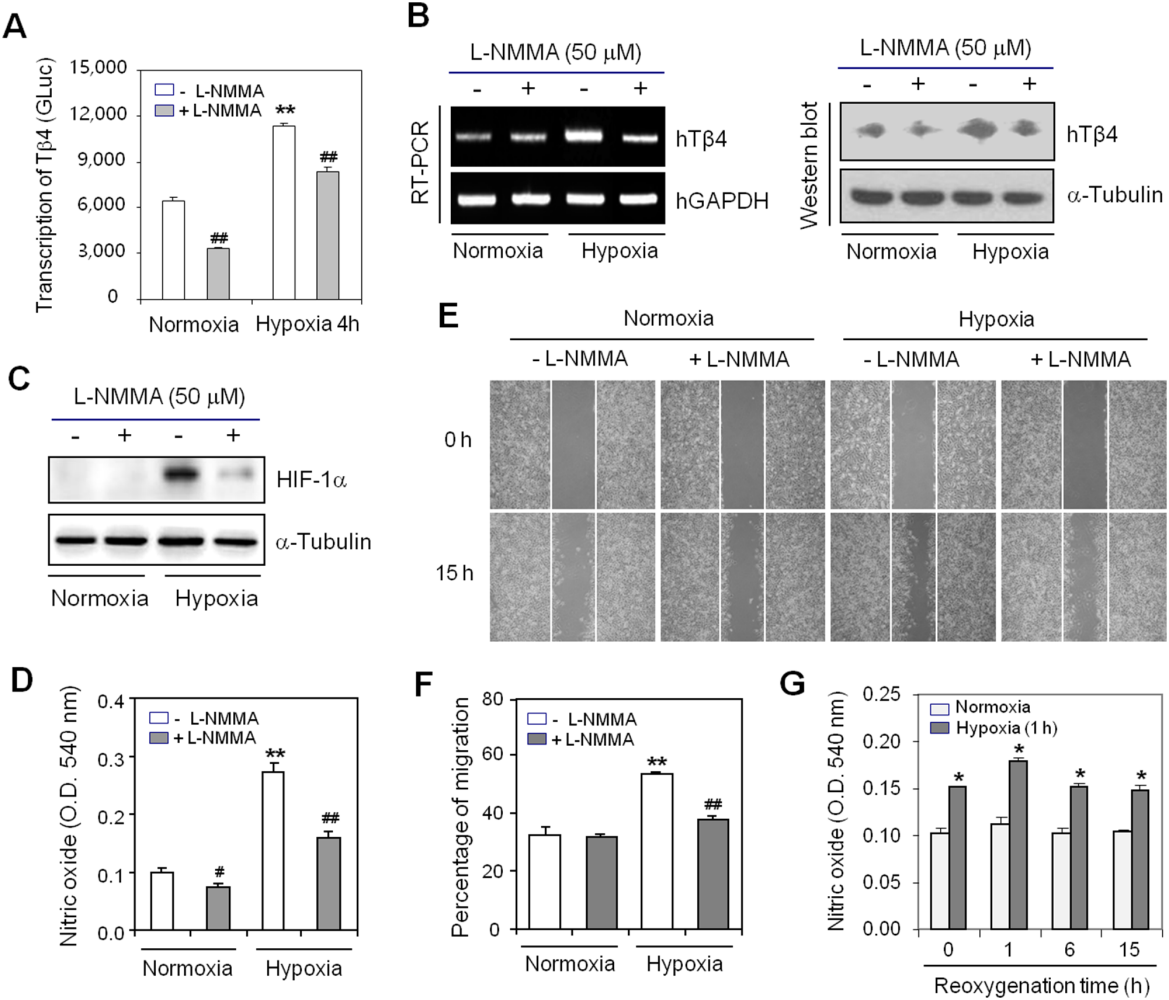
N^G^-monomethyl-L-arginine (L-NMMA), a NOS inhibitor, attenuated hypoxia-inducible Tβ4 expression and cell migration. **A:** HeLa cells were transfected with Tβ4-GLuc for 6 h and incubated under normoxic or hypoxic conditions for 4 h in the presence or absence of L-NMMA. GLuc activity in culture medium was measured using a luminometer with a GLuc substrate. Data in the bar graph are means ± SED. **p<0.01, statistical significance *vs*. Tβ4 promoter activity in the normoxia control group. ^##^ *p*<0.01, statistical significance *vs*. Tβ4 promoter activity in L-NMMA-untreated control at each normoxic or hypoxic condition. **B–C:** HeLa cells were incubated under normoxic or hypoxic conditions for 4 h in the presence or absence of L-NMMA. RNA was purified with TRIzol reagent and cell lysates were prepared as described in the Materials and Methods. The Tβ4 level was measured by RT-PCR (**B, left**) and Western blot analysis (**B, right**). HIF-1α was detected using Western blot analysis (**C**). **D:** NO production was detected with Griess reagents as described in the Materials and Methods. Data in the bar graph represent mean ± SED. **p<0.01, statistical significance *vs*. NO production in the normoxia control group. ^#^ *p*<0.05; ^##^ *p*<0.01, statistical significance *vs*. NO production in L-NMMA-untreated control at each normoxic or hypoxic condition. **E–G:** HeLa cells were scratched and incubated under normoxic conditions for 15 h in the presence or absence of L-NMMA after 1 h-incubation under normoxic or hypoxic condition. Cell migration into the space left by the scratch was photographed with a phase-contrast microscope. Photographs were taken at the same magnification; 200×. Data represent four experiments (**E**). The empty area was quantified using NIH image analysis software (version 1.62) and compared with the initiation of cell migration. Percentage of cell migration is presented as a bar graph. Data in the bar graph are means ± SED. **p<0.01, statistical significance *vs*. cell migration in the normoxia control group. ^##^ *p*<0.01, statistical significance *vs*. cell migration in the L-NMMA-untreated control under each normoxic or hypoxic condition (**F**). NO production was detected with Griess reagents as described in the Materials and Methods. Data in the bar graph represent mean ± SED. *p<0.05, statistical significance *vs*. NO production in the normoxia control group at each time point (**G**).

To confirm the effect of hypoxia-inducible NO on cell migration *via* Tβ4 expression in HeLa cells, we transfected HeLa cells with plasmid containing gaussia luciferase (GLuc) under the control of Tβ4 promoter and treated cells with L-NMMA that is a relatively non-selective inhibitor of all isoforms of nitric oxide synthase (NOS). Transcription of Tβ4 assessed by gaussia activity of Tβ4 promoter was reduced by the L-NMMA treatment under normoxic and hypoxic conditions (Fig. 3A). When HeLa cells were treated with L-NMMA under normoxic and hypoxic conditions, a decrease in Tβ4 expression was confirmed by RT-PCR (Fig. 3B, left) and Western blot analysis (Fig. 3B, right). The L-NMMA treatment also inhibited HIF-1α stabilization and NO production under the hypoxic condition (Fig. 3C and D). In addition, hypoxia conditioning-induced increase in cell migration was reversed with L-NMMA treatment (Fig. 3E and F). NO production was maintained in cells with hypoxia conditioning for 1 h and reoxygenation for 15 h as compared to hypoxia-unexperienced control cells (Fig. 3G). Data showed that NO-mediated Tβ4 expression might regulate hypoxia conditioning-induced cancer cell migration. It suggests that Tβ4 expression could be controlled by NO-mediated HIF-1α stabilization.

### Tβ4 expression was upregulated by NO-dependent HIF-1α

**Figure 4:**
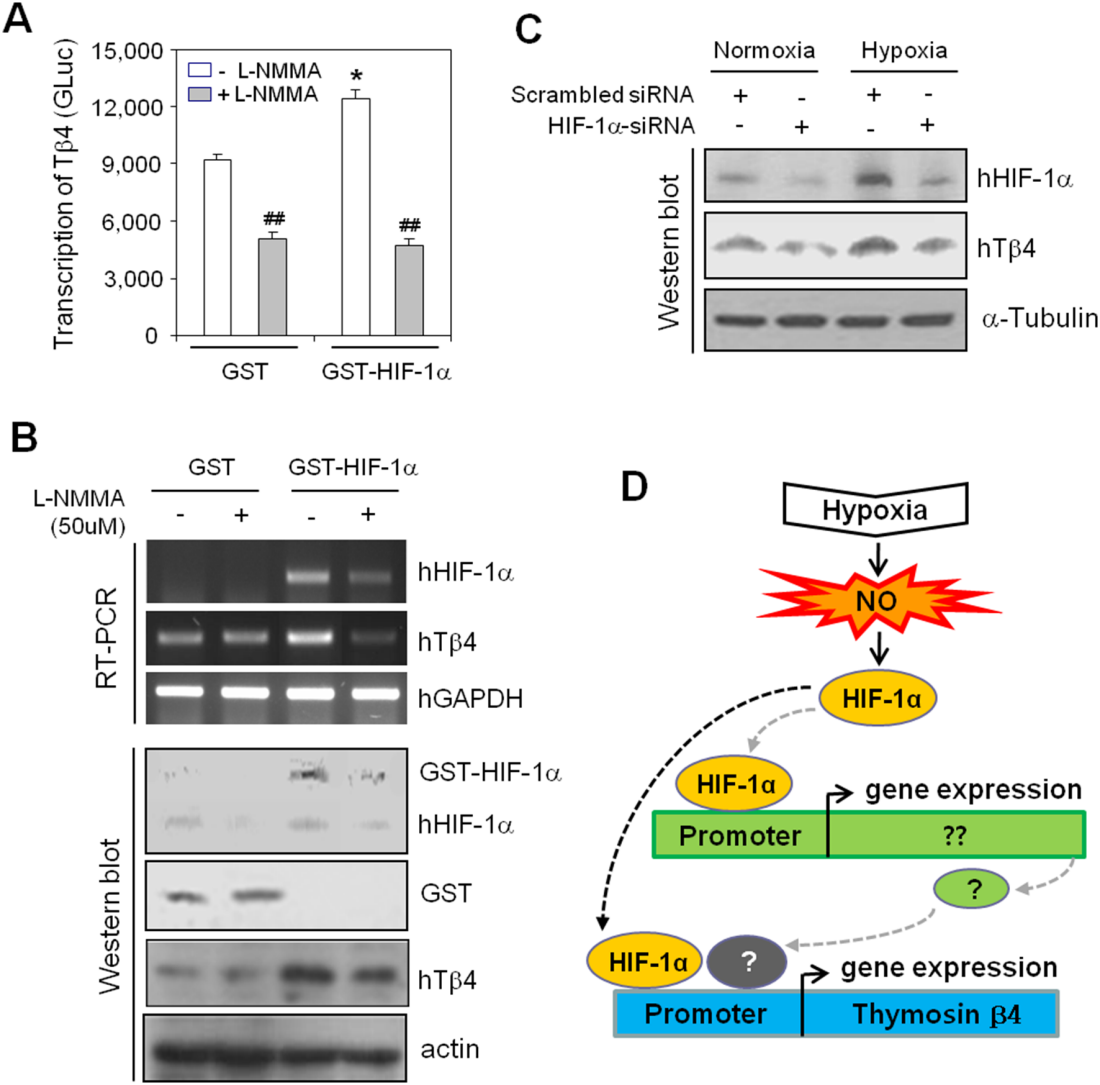
Tβ4 expression was increased by NO-associated HIF-1α expression. **A–B**: Tβ4-GLuc was co-transfected into HeLa cells with GST-HIF-1α and incubated in the presence or absence of L-NMMA. GLuc activity in culture medium was measured using a luminometer with a GLuc substrate. Data in the bar graph are means ± SED. *p<0.05, statistical significance *vs*. Tβ4 promoter activity in the GST vector-transfected control group. ^##^ *p*<0.01, statistical significance *vs*. Tβ4 promoter activity in the L-NMMA-untreated control for each GST- or GST-HIF-1α-transfected group (**A**). RNA for each group was purified with TRIzol reagent as described in the Materials and Methods. Tβ4 and HIF-1α transcript levels were measured using RT-PCR (**B, top**). Cell lysates were prepared from HeLa cells transfected with GST or GST-HIF-1α plasmid. Tβ4, GST, GST-HIF1α and HIF-1α protein levels were detected using Western blot analysis (**B, bottom**). **C:** HeLa cells were transfected with siRNA of HIF-1α gene for 24 h and incubated under normoxic or hypoxic conditions for 4 h. Tβ4 protein was detected using Western blot analysis. **D:** Scheme of possible mechanism underlying the effect on Tβ4 expression. Tβ4 could be a novel target of hypoxia-inducible NO and HIF-1α. Black and grey dotted arrows indicate direct and indirect effect of HIF-1α on Tβ4 expression, respectively. Question mark means unknown signaling molecules or transcription factors leading to an increase in Tβ4 expression.

To examine the role of NO-dependent HIF-1α on Tβ4 expression, the Tβ4 promoter was co-transfected into HeLa cells with glutathione transferase (GST)-HIF-1α and cells were incubated in the presence or absence of L-NMMA under normoxic condition. As shown in Figure 4A, transcription of Tβ4 assessed by gaussia activity of Tβ4 promoter was increased by co-transfection with GST-HIF-1α and its activity was reversed by addition of L-NMMA. Transcriptional and translational increase in Tβ4 expression was confirmed in the GST-HIF-1α-transfected group (Fig. 4B). To confirm HIF-1α-dependent Tβ4 expression, HIF-1α expression in HeLa cells was inhibited by the transfection with siRNA of HIF-1α gene. Data showed that Tβ4 expression was reduced by HIF-1α-siRNA in HeLa cells under normoxic or hypoxic conditions (Fig. 4C). These results showed that Tβ4 expression could be regulated by HIF-1α *via* its direct or indirect interaction with Tβ4 promoter (Fig. 4D). It suggests that HIF-1α-mediated Tβ4 expression might depend on NO production.

## Discussion

In cell migration, Tβ4 proteins regulate cancer cell migration under normoxic and hypoxic conditions [12] and by GSK-3α activation [13]. However, the regulation of Tβ4 expression in cancer cell migration has not been defined. Reportedly, NO production was increased in a hypoxic condition [23] and inhibited primary tumor growth. NO can also promote tumor invasiveness and metastasis [27], [28] by increasing cell motility in various types of cancer cells [28]–[30]. Herein, we investigated whether hypoxia-inducible NO could affect cancer cell migration, if NO could regulate Tβ4 expression in cancer cells, specifically in human cervical cancer cells. Our data showed that Tβ4 expression and cancer cell migration was increased by hypoxia-inducible NO and by the treatment with SNAP-1, a NO donor (Fig. 1). NO-induced cancer cell migration depended on Tβ4 expression (Fig. 2). The results were confirmed by L-NMMA treatment, a NOS inhibitor, which inhibited Tβ4 expression and cancer cell migration. L-NMMA treatment also inhibited hypoxia-induced cancer cell migration (Fig. 3). Next, Tβ4 transcriptional activity was enhanced by co-transfection with GST-conjugated HIF-1α and was inhibited by incubation in the presence of L-NMMA (Fig. 4). These results suggest that Tβ4 expression could be regulated by HIF-1α acting on the Tβ4 promoter *via* NO production in a hypoxic condition. In addition, it could not be ruled out more than one possibility for HIF-1α to increase Tβ4 activity by an increase in the expression of other promoter activator through indirect signaling pathway.

Previous reports showed that Tβ4 protein could lead in a tumor cell microenvironment to hypoxic condition enhancing HIF-1α stabilization [9]. Ac-SKDP is a bioactive fragment formed by enzymatic processing of the Tβ4 N-terminus [32], [33]. Ac-SDKP is also found in thymosin 11, 12, 14 and 15 [2]. HIF-1α stabilization and VEGF expression were reduced by the infection of Tβ4 lentiviral shRNA [12]. Although hypoxia-inducible NO led to an increase in Tβ4 expression *via* HIF-1α stabilization, the possibility that production of Ac-SKDP from Tβ4 could mediate HIF-1α stabilization leading to Tβ4 expression in an autocrine manner could not be eliminated.

According to previous reports, HIF-1α can be induced by many molecules such as guanylate cyclase as well as protein kinase C in aged skin [34], [35], p70S6K1 in prostate cancer cells [36] and PI3K/Akt in myotubes [37]. Reportedly, NO activates c-Src/PI3K- and PKG-dependent ERK 1/2 in insulin-producing RINm5F cells [38]. NO and cGMP stimulate p21Ras-Raf-1 kinase-MEK-ERK1/2 in rabbit aortic endothelial cells [39]. Therefore, the results observed with SNAP-1 treatment were likely due to the participation of other signaling molecules in induction of NO-mediated HIF-1α and consequently Tβ4 expression.

Phosphorylation by various kinases including PI3, ERK and p38 kinases activates HIF-1 [40]. ERK is activated in human microvascular endothelial cells-1 (HMEC-1) during hypoxia [41]–[43]. Tβ4 overexpression enhanced the basal level of phospho-Erk [44]. Therefore, Erk could control NO-induced HIF-1α, which should be clarified in further studies.

Collectively, although it remains to be clarified 1) whether a hypoxia response element is present in the promoter of Tβ4, 2) which sequence in the Tβ4 promoter binds HIF-1α, and 3) the mechanism of action underlying Tβ4 expression, hypoxia-inducible NO could influence the increase in Tβ4 expression in cervical cancer cells. Data suggest that NO may facilitate intracellular autocrine crosstalk between Tβ4 expression and HIF-1α induction indicating Tβ4 could be a novel target controlled by HIF-1α, potentially increasing cancer cell migration. It is also possible that HIF-1α could indirectly increase Tβ4 promoter activity not only by a direct acting on Tβ4 promoter but through an increase in the activity of other signaling molecules.

## Supporting Information

Figure S1 Hypoxia conditioning increased HeLa cell migration. **A–C:** A monolayer of HeLa cells was scratched and incubated under normoxic conditions for 15 h after 1 h-incubation under normoxic or hypoxic conditions. Then, each group was incubated for 15 h under normoxic conditions. Then, migration of cells into the space left by the scratch was photographed by phase contrast microscope. Pictures were taken at the same magnification; 200x. Data were the representative of four experiments **(A)**. Empty area was quantified with NIH image analysis software (version 1.62) and compared with that in the initiation of cell migration. Percentage of cell migration was represented with bar graph. Data in bar graph represent mean ± SED. **P<0.01, statistical significance vs. cell migration in control group without incubation under hypoxic conditions for 1 h **(B)**. Cell lysates were prepared and the protein level of HIF-1α and Tβ4 was detected using Western blot analysis **(C)**.
(TIF)
