# Supplementary figures and images for: The Actin-Sequestering Protein Thymosin Beta-4 Is a Novel Target of Hypoxia-Inducible Nitric Oxide and HIF-1α Regulation
Source: PLoS One. 2014 Oct 1;9(10):e106532. doi: 10.1371/journal.pone.0106532 (PMC4182666; doi:10.1371/journal.pone.0106532)

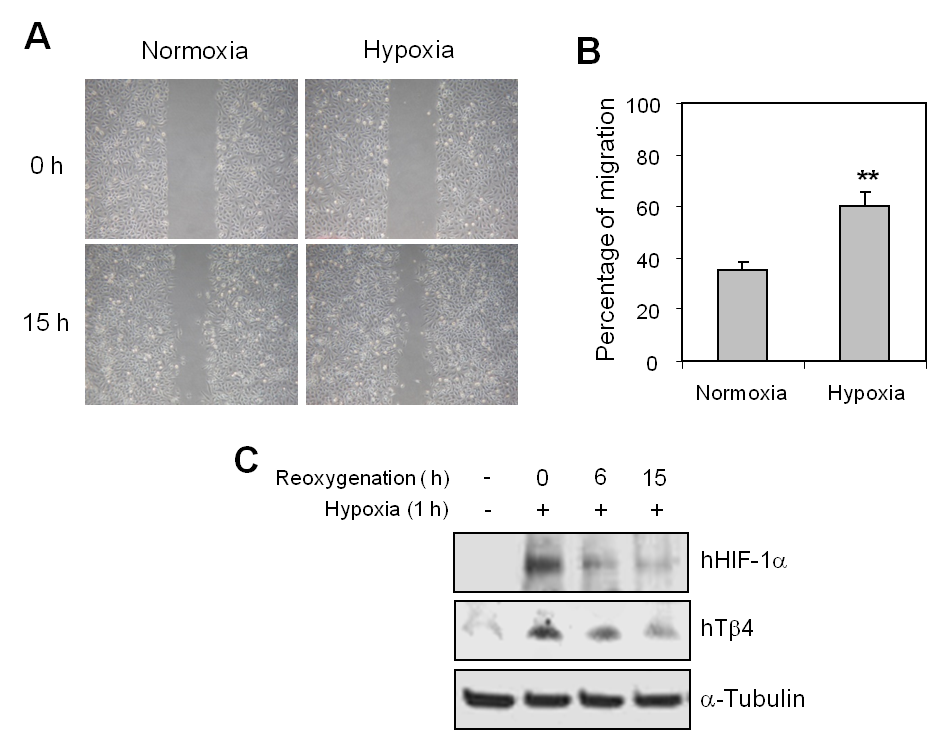

Supplement: Figure S1 — Hypoxia conditioning increased HeLa cell migration. A–C: A monolayer of HeLa cells was scratched and incubated under normoxic conditions for 15 h after 1 h-incubation under normoxic or hypoxic conditions. Then, each group was incubated for 15 h under normoxic conditions. Then, migration of cells into the space left by the scratch was photographed by phase contrast microscope. Pictures were taken at the same magnification; 200x. Data were the representative of four experiments (A). Empty area was quantified with NIH image analysis software (version 1.62) and compared with that in the initiation of cell migration. Percentage of cell migration was represented with bar graph. Data in bar graph represent mean ± SED. **P<0.01, statistical significance vs. cell migration in control group without incubation under hypoxic conditions for 1 h (B). Cell lysates were prepared and the protein level of HIF-1α and Tβ4 was detected using Western blot analysis (C). (TIF) [file pone.0106532.s001.tif]
